# Crucial Roles of Glu60 in Human Neuroglobin as a Guanine Nucleotide Dissociation Inhibitor and Neuroprotective Agent

**DOI:** 10.1371/journal.pone.0083698

**Published:** 2013-12-23

**Authors:** Nozomu Takahashi, Seiji Watanabe, Keisuke Wakasugi

**Affiliations:** Department of Life Sciences, Graduate School of Arts and Sciences, The University of Tokyo, Meguro-ku, Tokyo, Japan; Massachusetts General Hospital/Harvard Medical School, United States of America

## Abstract

Mammalian neuroglobin (Ngb) protects neuronal cells under conditions of oxidative stress. We previously showed that human Ngb acts as a guanine nucleotide dissociation inhibitor (GDI) for the α-subunits of heterotrimeric G_i/o_ proteins and inhibits reductions in cAMP concentration, leading to protection against cell death. In the present study, we created human E60Q Ngb mutant and clarified that Glu60 of human Ngb is a crucial residue for its GDI and neuroprotective activities. Moreover, we investigated structural and functional properties of several human Ngb mutants and demonstrated that the neuroprotective effect of human Ngb is due to its GDI activity and not due to its scavenging activity against reactive oxygen species.

## Introduction

Globins are iron porphyrin complex (heme)-containing globular proteins that bind reversibly to oxygen (O_2_) and, as such, play an important role in respiratory function. Mammalian neuroglobin (Ngb) is widely expressed in the brain [1]–[2]. The iron atom in the heme prosthetic group of Ngb normally exists in either the ferrous (Fe^2+^) or the ferric (Fe^3+^) redox state. Both the ferric and ferrous forms of Ngb are hexa-coordinated to endogenous protein ligands, namely proximal and distal His residues, and O_2_ displaces the distal His residue of ferrous Ngb to produce ferrous O_2_-bound Ngb [3]. The ferrous O_2_-bound form of Ngb that exists under normoxia is converted to the ferric conformation during oxidative stress, inducing large tertiary structural changes [4]. Mammalian Ngb proteins can protect neurons from hypoxic-ischemic insults and protect the brain from experimentally induced stroke *in vivo* [5]–[8].

Hypotheses of the neuroprotective mechanism of human Ngb have been reported previously [9]–[14]. Initially, Ngb was suggested to be an O_2_ storage protein [1]. However, the low concentration (in the micromolar range) of Ngb in brain tissues except for the retina perhaps argues against a role for Ngb in storing and carrying significant amounts of O_2_. Alternatively, Ngb may act as an intracellular scavenger of reactive oxygen species (ROS) and/or nitric oxide [15]–[19]. The reaction of ferric Ngb with hydrogen peroxide does not generate the highly reactive cytotoxic ferryl (Fe^4+^) species [18]. This property may be beneficial under conditions of oxidative stress.

To investigate other functions of human Ngb under conditions of oxidative stress, we previously performed yeast two-hybrid screening using human Ngb as a bait and identified flotillin-1, a lipid raft microdomain-associated protein, as a binding partner of human Ngb [20]. We demonstrated that human Ngb is recruited to lipid rafts by interacting with flotillin-1 only during oxidative stress and that lipid rafts are crucial for neuroprotection by Ngb [21]. We found that human ferric Ngb, which is generated under oxidative stress conditions, binds exclusively to the GDP-bound form of the α-subunits of heterotrimeric G_i/o_ proteins (Gα_i/o_), which is present in lipid rafts and inhibits adenylate cyclase activity [22], thereby acting as guanine nucleotide dissociation inhibitor (GDI) for Gα_i/o_ and inhibiting the reduction of intracellular cAMP concentration to protect against cell death [10], [21], [23]. By contrast, we previously showed that human ferrous ligand-bound Ngb under normoxia does not interact with Gα_i/o_ and does not have GDI activity [10], [21], [23]. We recently demonstrated that human Ngb acts as a non-receptor-mediated oxidative stress-responsive sensor for signal transduction in the brain [10], [21], [24].

**Figure 1:**
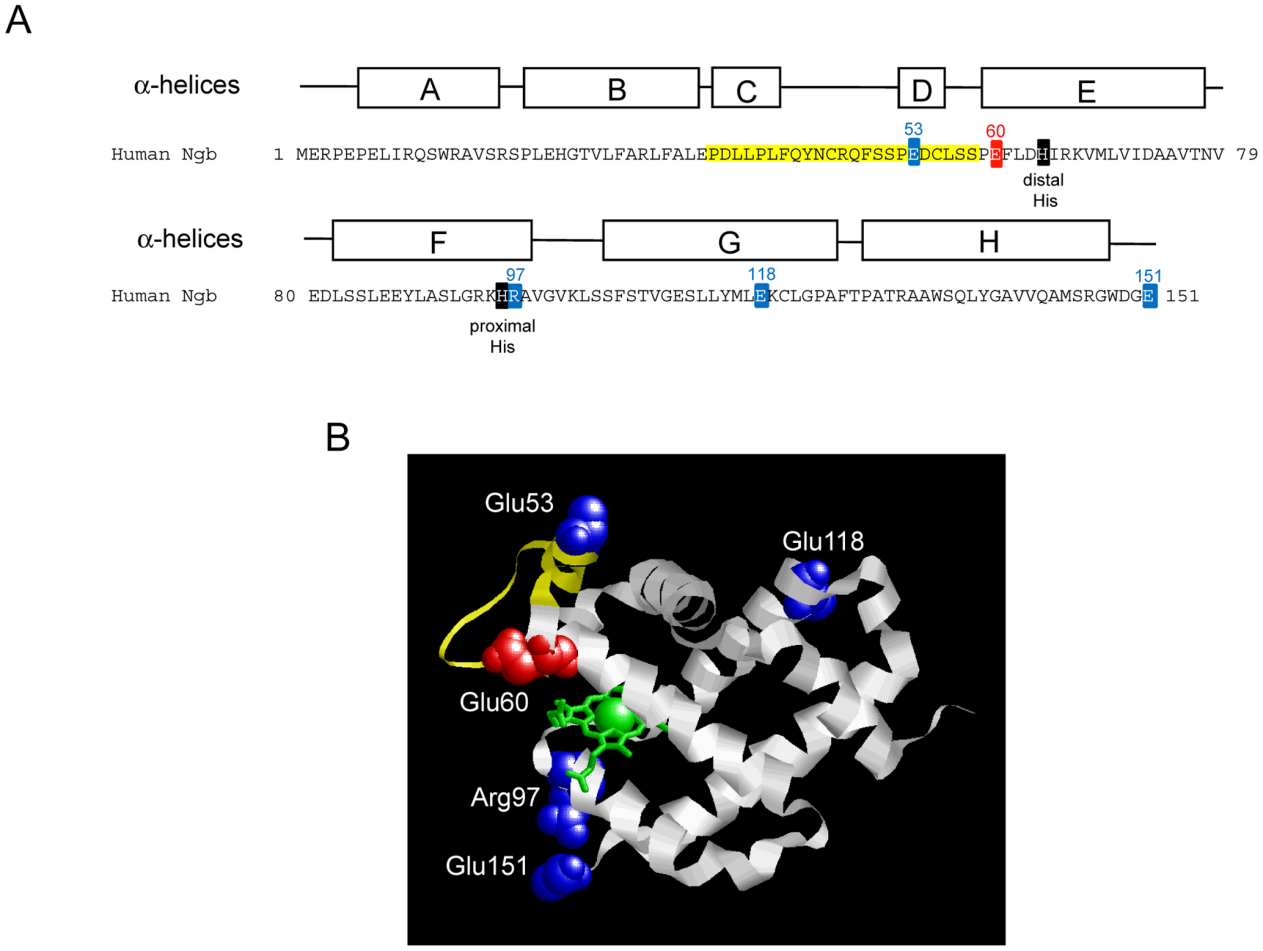
Structural position of Glu60 in human Ngb. (A) Sequence of human Ngb. The positions of α-helices A–H (Protein Data Bank Code: 1OJ6), and the proximal (His96) and distal (His64) histidine residues of human Ngb are shown. Glu60 is highlighted in red. Residues crucial for the GDI activity of human Ngb are marked in blue. Residues of CD-D region, composed by the α-helices C and D and the CD loop, are indicated in yellow. Numbers on the left and right of the sequences correspond to those at the beginning and end of the sequences, respectively. (B) Tertiary structure of human Ngb (Protein Data Bank code: 1OJ6). Glu60 is highlighted in red. Residues in human Ngb crucial for its GDI activity are indicated in blue. Residues of the CD-D region are indicated in yellow. Heme is indicated in green.

Although Ngb was originally identified in mammalian species, it is also present in non-mammalian vertebrates [25], [26]. We found that zebrafish Ngb does not exhibit GDI activity [27]. In order to clarify residues of human Ngb that are crucial for its GDI activity, we prepared human Ngb mutants with a focus on residues differing between human and zebrafish Ngb and on exposed residues with positive or negative charges on the protein surface [27]. We showed that human E53Q, R97Q, E118Q, and E151N Ngb mutants, which did not function as GDI proteins, did not rescue cell death under oxidative stress conditions [8], [27], indicating that Glu53, Arg97, Glu118 and Glu151 of human Ngb are crucial residues for its GDI activity and that the GDI activity of human wild-type (WT) Ngb is tightly correlated with its neuroprotective activity. Furthermore, Matrix-assisted laser desorption/ionization time-of-flight (MALDI-TOF) mass spectrometry (MS) analysis of tryptic peptides derived from a cross-linked complex between human WT Ngb and Gα_i1_, which is a member of the Gα_i/o_ family [22], revealed cross-linking between Glu60 (Ngb) and Ser206 (Gα_i1_), and between Glu53 (Ngb) and Ser44 (Gα_i1_) [23]. Glu53 as well as Arg97, Glu118 and Glu151 of Ngb are conserved only among boreotheria mammals [28], whereas Glu60 is highly conserved throughout vertebrates. As shown in Figs. 1A and 1B, Glu53 and Glu60 of human Ngb are located in and near the CD-D region, where large tertiary structural changes are induced by the conversion of ferrous O_2_-bound Ngb to ferric Ngb during oxidative stress [4].

In the present study, to clarify the role of Glu60 of human Ngb upon neuroprotective activity, we created human E60Q Ngb mutant. We clarified that the Glu60 of human Ngb is a crucial residue for its GDI and neuroprotective activities. Moreover, we investigated the structure and ROS scavenging activities of human E53Q, R97Q, E118Q, and E151N Ngb mutants as well as the E60Q mutant and demonstrated that the neuroprotective effect of human WT Ngb is due to its GDI activity and not due to its scavenging activities against ROS.

## Materials and Methods

### Reagents

[8,5’-^3^H]GDP (20-50 Ci/mmol) was purchased from PerkinElmer Life Sciences (Boston, MA). Horse heart myoglobin (Mb) and N-acetylcysteine (NAC) were obtained from Sigma-Aldrich (St. Louis, MO) and Wako Pure Chemical Industries (Osaka, Japan), respectively.

### Preparation of recombinant human Ngb proteins

Plasmids for human Ngb were prepared as described previously [10], [27]. A QuikChange^TM^ site-directed mutagenesis system (Stratagene, La Jolla, CA) was used for site-directed mutagenesis. The constructs were confirmed by DNA sequencing (FASMAC Co., Ltd., DNA sequencing services, Atsugi, Japan). Overexpression of each Ngb was induced in *E. coli* strain BL 21 (DE 3) by treatment with isopropyl-β-D-thiogalactopyranoside (IPTG) for 4 h, and each Ngb protein was purified as described previously [8], [10], [21], [27]. In brief, soluble cell extracts were loaded onto DEAE sepharose anion-exchange columns equilibrated with buffer A (20 mM Tris-HCl, pH 8.0). Ngb proteins were eluted from columns with buffer A containing 150 mM NaCl, and further purified by passage through Sephacryl S-200 HR gel filtration columns. Ngb proteins were then applied to a HiTrap Q HP column (GE Healthcare Biosciences, Piscataway, NJ), eluted with a 0–500 mM linear NaCl gradient in buffer A. Purified Ngb was dialyzed overnight against phosphate-buffered saline (PBS). Endotoxin was removed from the protein solutions by phase separation using Triton X-114 (Sigma-Aldrich) [29], [30]. Trace amounts of Triton X-114 were removed by passage through Sephadex G25 gel (GE Healthcare Biosciences) equilibrated with PBS. The protein concentration of human ferric Ngb was determined spectrophotometrically using an extinction coefficient of 122 mM^−1^cm^−1^ at the Soret peak.

### UV-visible spectra

Electronic absorption spectra of purified proteins were recorded with a UV-visible spectrophotometer (UV-2450; Shimadzu, Kyoto, Japan) at ambient temperature (∼20°C). Spectra were recorded in PBS (pH 7.4).

### Circular dichroism (CD) spectra

CD spectra in the far UV region were measured with a spectropolarimeter (J-805; JASCO Co., Tokyo, Japan) at 20°C. The samples were measured at a concentration of approximately 5 µM in 50 mM sodium phosphate buffer (pH 7.4). The path length of the cells used for the measurements was 1 mm. The molar ellipticity (deg cm^2^ dmol^−1^) was determined on the mean residue basis. The α-helix content (*f*_H_) was calculated according to Chen et al. [31] by the following equation: 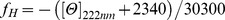



### Denaturation assays

Guanidine hydrochloride (GdnHCl)-induced denaturation experiments were carried out in 50 mM sodium phosphate buffer (pH 7.4), containing various concentrations of GdnHCl. The solutions contained 5 µM protein and were incubated for at least 4 h. CD spectra from 200 to 250 nm were measured. The fractional denatured population (*f*_D_) under each condition was estimated by the following equation: 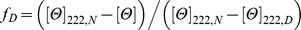



where [*Θ*]_222,N_, [*Θ*]_222,D_, and [*Θ*] represent ellipticities at 222 nm in the native and denatured states, and under each GdnHCl concentration, respectively.

The free energy of denaturation, Δ*G*, was calculated by the following equation: 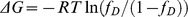



When Δ*G* varies linearly with the GdnHCl concentration, [GdnHCl], Δ*G*_H2O_, extrapolated to Δ*G* at [GdnHCl]  =  0, can be estimated by the following equation: 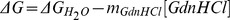



where *m*_GdnHCl_ is the slope of the linear relation between Δ*G* and [GdnHCl].

### Preparation of recombinant human Gα_i1_ protein

The DNA fragment containing the human Gα_i1_ subunit (residues 1–354) was amplified by PCR and cloned into the pET151/D-TOPO® vector (Invitrogen, Carlsbad, CA) to be expressed as human WT Gα_i1_ protein fused to a TEV protease recognition site directly after an N-terminal tag of six histidine residues (His_6_-tag) [21]. The resulting Gα_i1_ was expressed in *E. Coli* strain BL21 (DE3) by induction with IPTG and purified by using a nickel affinity column (His·Bind® resin; Novagen, Madison, WI), as described in [21]. Then, the sample was incubated with His_6_-tagged TEV protease (MoBiTec GmbH, Göttingen, Germany) and loaded onto a His·Bind® column to separate the cleaved Gα_i1_ from the cleaved His_6_-tag, any uncleaved protein, and His_6_-tagged TEV protease, as described in [21].

### [^3^H]GDP dissociation assays

GDP dissociation assays were performed, as described in [21]. In brief, Gα_i1_ complexed with [^3^H]GDP (0.3 µM) was prepared by incubating 0.3 µM Gα_i1_ with 2 µM [^3^H]GDP in buffer B [20 mM Tris-HCl, 100 mM NaCl and 10 mM MgSO_4_ at pH 8.0] for 1.5 h at 25°C. Excess unlabeled GTP (2 mM) was added to monitor dissociation of [^3^H]GDP from Gα_i1_ in the absence or presence of Ngb (10 µM). Aliquots were withdrawn at 0, 5, and 10 min and were passed through nitrocellulose filters (0.45 µm) (Advantec Toyo, Tokyo, Japan). The filters were then washed three times with 1 ml of ice-cold buffer B and were counted in a liquid scintillation counter (LS6500; Beckman Coulter, Fullerton, CA).

### Cell culture

SH-SY5Y cells (CRL-2266) were obtained from the American Type Culture Collection (ATCC; Manassas, VA) and maintained in a 1∶1 mixture of Dulbecco’s modified Eagle’s medium (DMEM) and Ham’s F-12 nutrient mixture containing 2.5 mM glutamine, supplemented with 10% (v/v) fetal bovine serum (FBS), 100 U/ml penicillin, and 100 µg/ml streptomycin (all from Invitrogen) in a humidified atmosphere containing 5% CO_2_ at 37°C. The medium was changed every 4 days, and the cultures were split at a 1∶20 ratio once a week. Cultured cells were induced to differentiate into a neuronal phenotype by treatment with 10 µM retinoic acid (Sigma-Aldrich) over a period of 6 days (media were exchanged every 3 days during sub-culture). Differentiation was verified by monitoring macroscopic changes to the cells.

### Protein transduction by Chariot

Protein transduction was performed by using Chariot™ (Active Motif, Carlsbad, CA) as described previously [8], [21]. Each purified Ngb protein (3 µg per well) was incubated in the presence of diluted Chariot for 30 min at room temperature. Next, the mixture was added to differentiated SH-SY5Y cells that had been washed in DMEM without serum. DMEM without serum was added and the cells were incubated at 37°C for 1 h; FBS was then added to a final concentration of 2%. The cells were incubated at 37°C for another 2 h to allow Ngb internalization.

### Oxygen-glucose deprivation (OGD)

The day before experiments, differentiated SH-SY5Y cells were plated on a poly-D-lysine-coated 96-well tissue culture plate at a density of 5×10^5^ cells / mL. Protein transduction was performed by Chariot™. An *in vitro* model of ischemia was created by maintaining SH-SY5Y cells under OGD for 16 h, followed by 24 h of recovery. In brief, the standard culture medium was replaced with a glucose-free OGD buffer (154 mM NaCl, 5.6 mM KCl, 5.0 mM HEPES, 3.6 mM NaHCO_3_, and 2.3 mM CaCl_2_ at pH 7.4). Hypoxia was induced in a multi-gas incubator (Astec, Fukuoka, Japan; set to 1% O_2_, with 5% CO_2_ and 94% N_2_) at 37°C for 16 h. After hypoxia, the culture medium was replaced with standard culture medium ( a 1 : 1 mixture of Dulbecco’s modified Eagle’s medium and Ham’s F-12 containing 2.5 mM glutamine, supplemented with 10% (v/v) fetal bovine serum), and the cells were incubated at 37°C for 24 h under normoxia (95% air/5 % CO_2_).

### Transfection of human Ngb expression vector into SH-SY5Y cells and treatment of cells with hydrogen peroxide

The eukaryotic expression vector pcDNA3.1 (Invitrogen) for human Ngb was prepared as described previously [21]. A QuikChange^™^ site-directed mutagenesis system (Stratagene, La Jolla, CA) was used to introduce the E60Q substitution and the construct was confirmed by DNA sequencing (FASMAC Co., Ltd., DNA sequencing services). Differentiated SH-SY5Y cells were plated on poly-D-lysine coated 96-well plates at a density of 5.0×10^5^ cells/mL for 24 h. The pcDNA3.1-human WT or E60Q Ngb expression vector or control vector (pcDNA3.1 empty vector) was transfected by using Lipofectamine™ 2000 (Invitrogen) according to the manufacturer’s instructions. After 24 h of transfection, hydrogen peroxide was added at 100 µM and cells were incubated for 24 h.

### Western blot analyses

Protein transduction or transfection of expression vector was confirmed by Western blot analyses using rabbit anti-Ngb (FL-151) polyclonal antibody (Santa Cruz Biotechnology, Santa Cruz, CA), mouse anti-β-actin monoclonal antibody (Sigma-Aldrich). After washing, membranes were incubated with an HRP-linked F(ab’)_2_ fragment of donkey anti-rabbit IgG or an HRP-linked whole antibody of sheep anti-mouse IgG (GE Healthcare Biosciences). Proteins were visualized using ECL™ western blotting detection reagents (GE Healthcare Biosciences). Chemiluminescent signals were detected using a LAS-4000 mini luminescent image analyzer (GE Healthcare Biosciences).

### Cell viability assays

Cell viability was measured by trypan blue exclusion (TBE) assays. Trypan blue was added to the cultured cells, and the percentage of blue-stained cells was calculated after counting at least 1000 cells via phase contrast microscopy. Cell viability was also measured with the CellTiter 96® AQueous One Solution Cell Proliferation Assay Reagent (Promega, Madison, WI), containing [3-(4,5-dimethylthiazol-2-yl)-5-(3-carboxymethoxyphenyl)-2-(4-sulfophenyl)-2H-tetrazolium, inner salt; MTS]. Cultured cells were incubated with the MTS reagent at 37°C for 4 h in a humidified, 5 % CO_2_ atmosphere. The amount of colored formazan dye formed was then quantified by measuring absorbance at 490 nm with a Beckman Coulter DTX880 plate reader (Beckman Coulter, Fullerton, CA).

### Hydroxyl radical scavenging assay

The hydroxyl radical scavenging activities were measured and analyzed, as described previously [19], [32]. In this system, hydroxyl radicals were generated by the Fenton reaction. Briefly, the reaction mixture included 100 µL of 0.75 mM 1,10-phenanthroline, 200 µL of 5 mM PBS (pH 7.2), 100 µL of 0.75 mM FeSO_4_, 50 µL of 0.01% hydrogen peroxide, and 50 µL of Milli-Q-purified water. The reaction was initiated by addition of hydrogen peroxide. After incubation at 37°C for 60 min, the absorbance of the mixture at 536 nm was measured, as *A*_f_. The hydroxyl radical scavenging activity was calculated by the following equation:
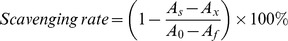



where *A*_0_ is the absorbance using Milli-Q-purified water instead of hydrogen peroxide, *A*_s_ is the absorbance using sample (100 µg/mL) instead of hydrogen peroxide, and *A*_x_ is the absorbance using sample (100 µg/mL) instead of Milli-Q-purified water.

## Results

### Structural analyses of human E60Q Ngb mutant

**Figure 2:**
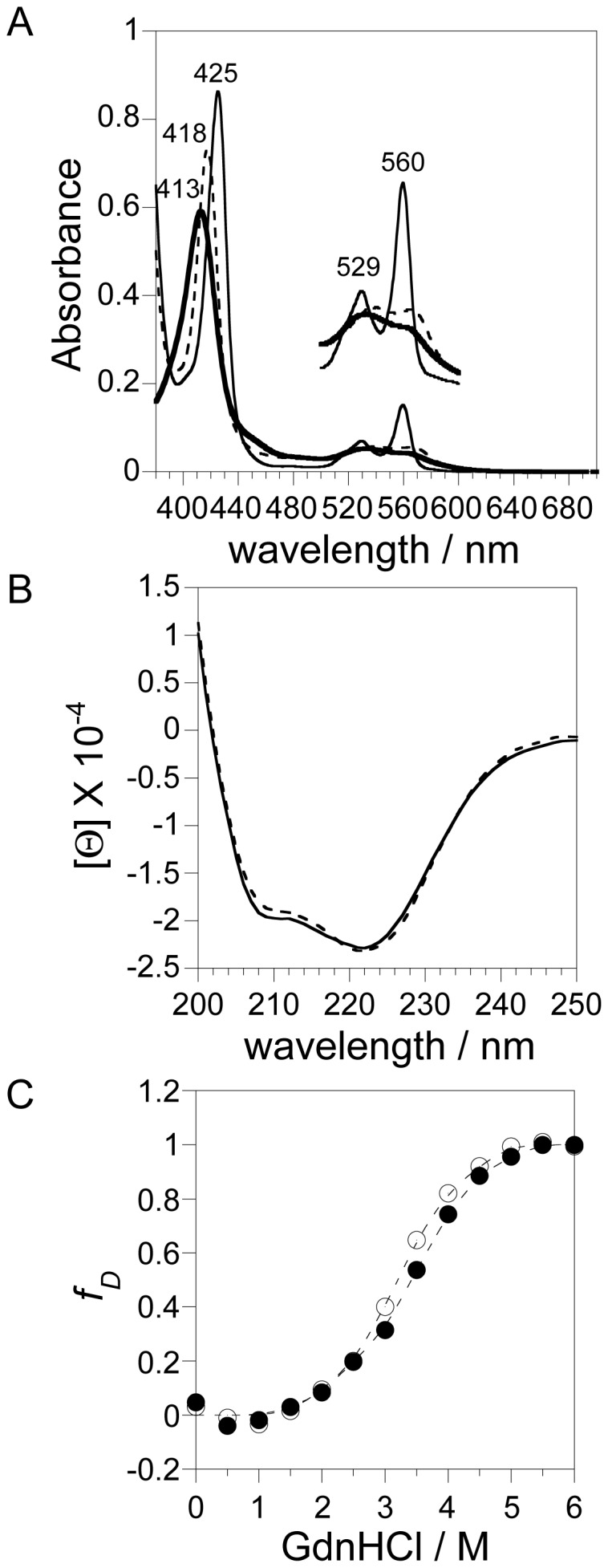
Effect of the E60Q mutation in human Ngb upon structure. (A) Electronic absorption spectra of the ferric (bold line), ferrous deoxy (fine line), and ferrous CO-bound (dotted line) forms of human E60Q Ngb. The Q bands from 500 to 600 nm are enlarged by a factor of 3 on the perpendicular axis. The spectra were recorded in PBS (pH 7.4) at ambient temperature (∼20°C). (B) CD spectra in the far UV region of the ferric form of the WT Ngb (dotted line), and E60Q Ngb (fine line). The concentration of each protein was approximately 5 µM on the basis of heme content. The spectra were recorded in 50 mM sodium phosphate buffer (pH 7.4) at 20°C. (C) GdnHCl denaturation curves of human WT and E60Q Ngb. Denaturation curves were measured for human WT (open circle), and E60Q (closed circle) Ngb. Molecular ellipticities at 222 nm in the native and completely denatured states were normalized to 0 and 1, respectively. The concentration of each protein was 5 µM on the basis of heme content.

Initially, we evaluated the effects of the E60Q mutation on the electronic state of the heme group by measuring the absorption spectra of the E60Q Ngb mutant. Fig. 2A shows the UV-visible spectra of the ferric, ferrous deoxy, and ferrous carbon monoxide (CO)-bound forms of human E60Q Ngb. Because ferrous O_2_-bound Ngb is unstable and is converted into ferric Ngb very rapidly due to autoxidation [3], stable ferrous CO-bound Ngb was used as a model for ferrous O_2_-bound Ngb. The wavelengths of the Soret peaks of human ferric, ferrous deoxy, and ferrous CO-bound E60Q Ngb were 413, 425, and 418 nm, which were the same as those of human WT Ngb (413, 425, and 418 nm), respectively [33], demonstrating that the E60Q mutation of human Ngb did not perturb the electric state of the heme group. Moreover, the absorption ratio of the Soret peak and at 280 nm suggested that the Ngb mutant bind heme just as tightly as the WT protein (Table 1).

**Table 1:** Structural data of human ferric WT Ngb and Ngb mutants of crucial residues for protein-protein interaction between Gα_i1_ and Ngb.

	WT	E53Q	E60Q	R97Q	E118Q	E151N
*Electronic absorption spectra*						
Soret (nm)	413	413	413	413	413	413
visible (nm)	533	532	534	532	532	532
A_Soret_/A_280nm_	2.80	2.51	2.81	2.38	2.58	2.90
*CD spectra*						
[*Θ*]_222nm_x (10^−4^) (deg cm^2^ dmol^−1^)	–2.32	–2.40	–2.29	–2.26	–2.34	–2.36
α-helical content (%)	68.9	71.5	67.9	66.9	69.5	70.2
*m*_GdnHCl_ (kJ mol^−1^ M^−1^)	20.2	20.4	19.2	19.3	20.0	19.8
Δ*G*_H2O_ (kJ mol^−1^)	63.8	65.5	63.6	63.4	61.6	64.6

Next, to examine the effect of the E60Q substitution upon secondary structure, we measured the far UV CD spectra of the ferric forms of human WT or E60Q Ngb. As shown in Fig. 2B, human WT and E60Q Ngb proteins exhibited two negative broad peaks around 222 and 208 nm, which are characteristic of an α-helical structure. The α-helical content of the E60Q Ngb protein was estimated to be 67.9%, which is almost identical to that of human WT Ngb (68.9%) (Table 1). These results showed that the secondary protein structure is not affected by the amino acid substitution.

Alterations in equilibrium stability caused by the E60Q substitution were quantified in a GdnHCl-induced denaturation experiment. The GdnHCl denaturation process was followed by monitoring the ellipticity value at 222 nm, which reflects structural changes in the whole protein. As shown in Fig. 2C, the ferric forms of WT and E60Q Ngb showed the cooperative transition curves. The transition curve for GdnHCl denaturation of E60Q Ngb was similar to the curves for human WT Ngb, indicating that the globular structure of E60Q Ngb was as stable as those of the WT Ngb.

### Glu60 of human Ngb is a crucial residue for its GDI and neuroprotective activities

**Figure 3:**
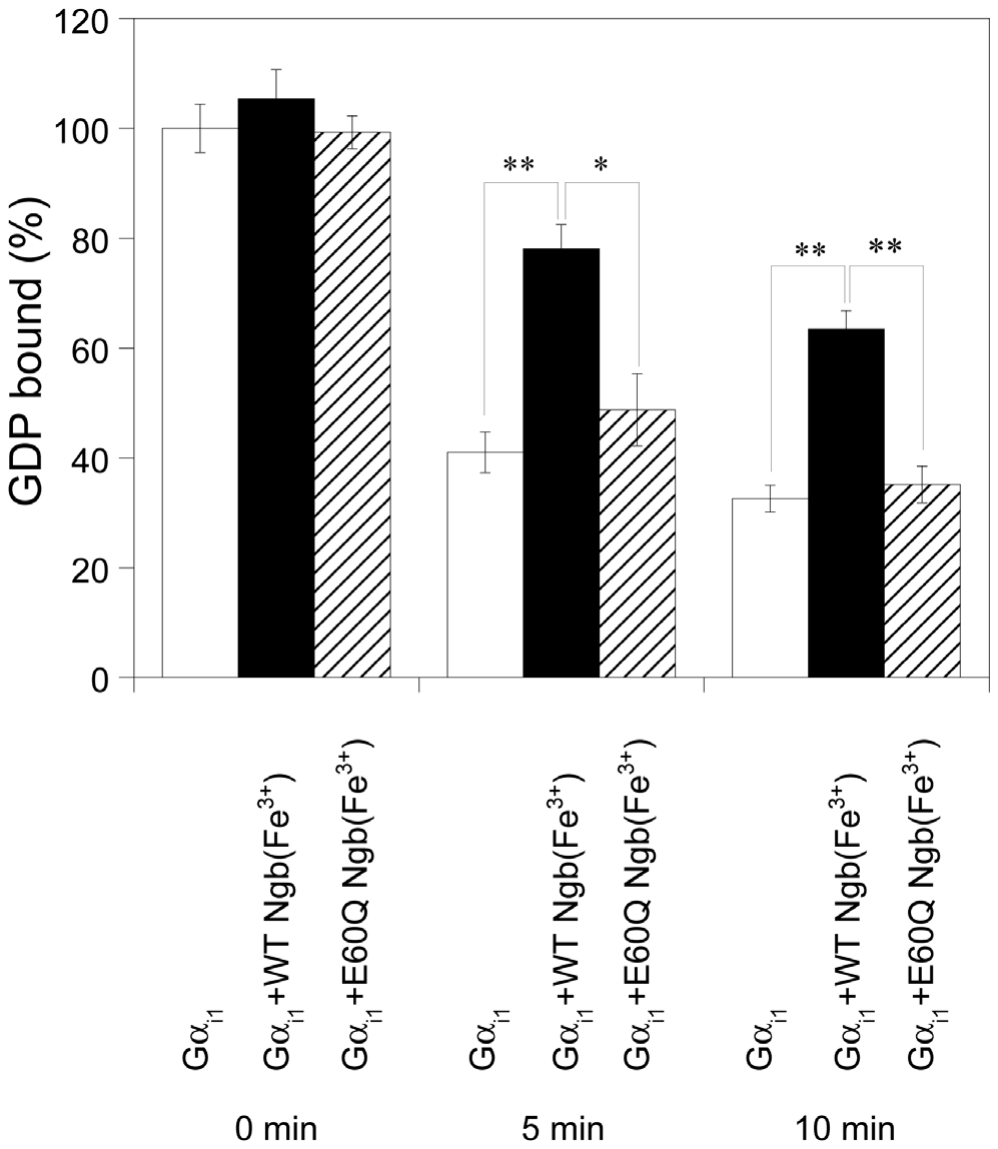
Effects of human WT Ngb or E60Q Ngb on the dissociation of GDP from human GDP-bound Gα_i1_. The amount of [^3^H]GDP bound to Gα_i1_ in the absence of human Ngb at 0 min was defined as 100%. All data are expressed as means ± standard error of means (SEM) from four independent experiments. Data were analyzed by one-way ANOVA followed by Tukey-Kramer post hoc tests. *P<0.05, **P<0.01.

To examine the effect of the E60Q mutation of human Ngb upon the release of GDP from Gα_i1_, we measured the rates of GDP dissociation in the absence or presence of human Ngb. As shown in Fig. 3, [^3^H]GDP release from [^3^H]GDP-bound Gα_i1_ was inhibited by human ferric WT Ngb in the presence of an excess amount of unlabeled GTP. By contrast, human ferric E60Q Ngb did not function as the GDI for Gα_i1_ (Fig. 3).

**Figure 4:**
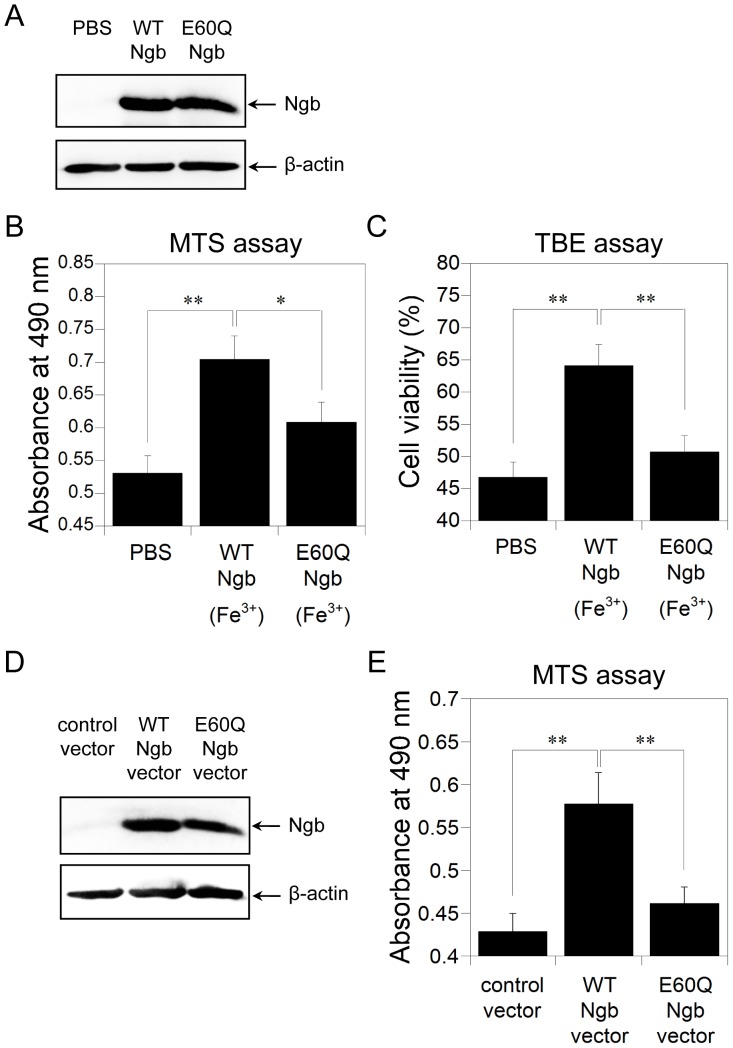
Effects of human WT Ngb or E60Q Ngb on neuroprotective activities. (A) Western blot analyses of SH-SY5Y cell lysates after protein transduction. PBS, human WT Ngb or human E60Q Ngb was applied to differentiated SH-SY5Y cells with Chariot. The cells were then incubated for 3 h. Cell lysates were analyzed on 15.0% or 12.5% SDS/PAGE and by Western blot analyses using rabbit anti-Ngb polyclonal antibody or mouse anti-β-actin monoclonal antibody, respectively. (B,C) Effect of the E60Q mutation in human Ngb on SH-SY5Y cell death caused by the *in vitro* OGD model of ischemia. Human WT or E60Q Ngb was transfected into differentiated cells using Chariot. Following OGD/recovery, cell viability was measured by MTS (B) and TBE assays (C). All data are expressed as means ± SEM from three independent experiments, each performed in triplicate. Data were analyzed by one-way ANOVA followed by Tukey-Kramer post hoc tests. *P<0.05, **P<0.01. (D) Western blot analyses of SH-SY5Y cell lysates after transfection. Control vector, human WT or E60Q Ngb expression vector was transfected into differentiated SH-SY5Y cells with Lipofectamine. The cells were then incubated for 24 h. Cell lysates were analyzed on 15.0% or 12.5% SDS/PAGE and by Western blot analyses using rabbit anti-Ngb polyclonal antibody or mouse anti-β-actin monoclonal antibody, respectively. (E) Effect of the E60Q mutation in human Ngb on SH-SY5Y cell death caused by hydrogen peroxide. Differentiated SH-SY5Y cells transfected with control vector, human WT or E60Q HNgb expression vector with Lipofectamine were treated with hydrogen peroxide, and cell viability was measured by MTS assay. All data are expressed as means ± SEM from six independent experiments, each carried out in triplicate. Data were analyzed by one-way ANOVA followed by Tukey-Kramer post hoc tests. **P<0.01.

Next, we used SH-SY5Y cells differentiated into a neuron-like type to evaluate whether human Ngb protects cells against ischemia in an *in vitro* OGD model of *in vivo* ischemia-reperfusion insult. Protein transduction was achieved by using the protein delivery reagent Chariot, which can efficiently deliver a variety of proteins into several cell lines in a fully biologically active form [8], [21], [34]–[36], and was confirmed by Western blot analyses (Fig. 4A). MTS assays showed that cell survival was significantly enhanced by the transduction of human WT Ngb into SH-SY5Y cells (Fig. 4B). TBE assays also showed that protein transduction of human WT Ngb via Chariot resulted in a significant increase in cell viability (Fig. 4C). These results suggest that human WT Ngb is effective in rescuing SH-SY5Y cell death induced by the OGD model. By contrast, the E60Q Ngb mutant, which lacked GDI activity, did not significantly rescue cell death under oxidative stress conditions (Figs. 4B and 4C). Moreover, the protective effect of human WT Ngb, but not human E60Q Ngb, was also confirmed by the following experiment: a pcDNA3.1-human WT or E60Q Ngb expression vector, or a control vector (pcDNA3.1 empty vector) was transfected into SH-SY5Y cells by Lipofectamine and the protective effects of Ngb proteins against hydrogen peroxide-induced cell death was tested. Expression of human Ngb proteins was confirmed by Western blot analyses (Fig. 4D). As shown in Fig. 4E, MTS assays showed that human WT Ngb enhanced cell survival. By contrast, human E60Q Ngb did not protect SH-SY5Y cells against cell death (Fig. 4E). Taken together, we conclude that the Glu60 of human Ngb is crucial for its GDI and neuroprotective activities.

### The neuroprotective effect of human WT Ngb is not due to its scavenging activities against ROS

We previously showed that human E53Q, R97Q, E118Q, and E151N Ngb mutants, which do not function as GDI proteins, do not rescue cell death under oxidative stress conditions [8], [27]. In the present study, we evaluated the effects of these mutations on the electronic state of the heme group and on the secondary structures of the ferric form of the Ngb mutants by measuring the UV-visible spectra and the far UV CD spectra, respectively. The absorption spectra of human Ngb mutants were nearly identical to, and the α-helical contents were almost the same as, that of human WT Ngb (Table 1). Alterations in equilibrium stability caused by the substitutions were quantified in a GdnHCl-induced denaturation experiment. The denaturation transition curves were fitted by a single two-state model to obtain the thermodynamic parameters of Ngb. As shown in Table 1, *m*_GdnHCl_ values and Δ*G*_H2O_ values of human Ngb mutants were almost the same as that of human WT Ngb, indicating that the globular structures of Ngb mutants were as stable as that of the wild-type Ngb. These data verified that these mutations do not induce significant structural changes, indicating that Glu53, Arg97, Glu118, and Glu151 are crucial residues for the GDI and neuroprotective activities of human Ngb.

**Figure 5:**
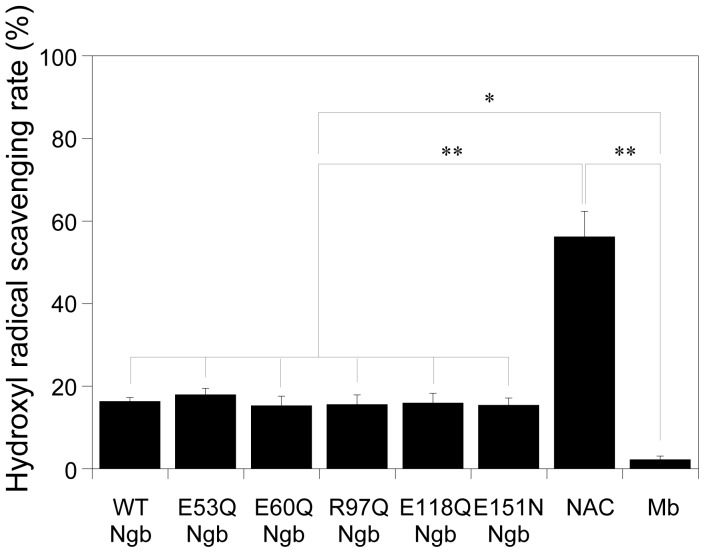
Hydroxyl radical scavenging activities of human WT, E53Q, E60Q, R97Q, E118Q, E151N Ngb, a ROS scavenger, N-acetylcysteine (NAC), and myoglobin (Mb). The reaction was initialized by addition of hydrogen peroxide and was incubated at 37°C for 60 min. The absorbance of the mixture at 536 nm was measured. Data are presented as the mean ± SEM of three experiments. Data were analyzed by one-way ANOVA followed by Tukey-Kramer post hoc tests. *P<0.05, **P<0.01.

In order to gain further insight into the neuroprotective mechanism of Ngb under conditions of oxidative stress, we measured the scavenging activities against the hydroxyl radical, which is the most reactive oxidant among ROS. Our data clearly showed that hydroxyl radical scavenging activities of human Ngb mutant proteins are almost identical to that of the WT Ngb and are much higher than that of Mb (Fig. 5). These results suggest that the neuroprotective effects of human WT Ngb are not due to their scavenging activities against ROS.

## Discussion

**Figure 6:**
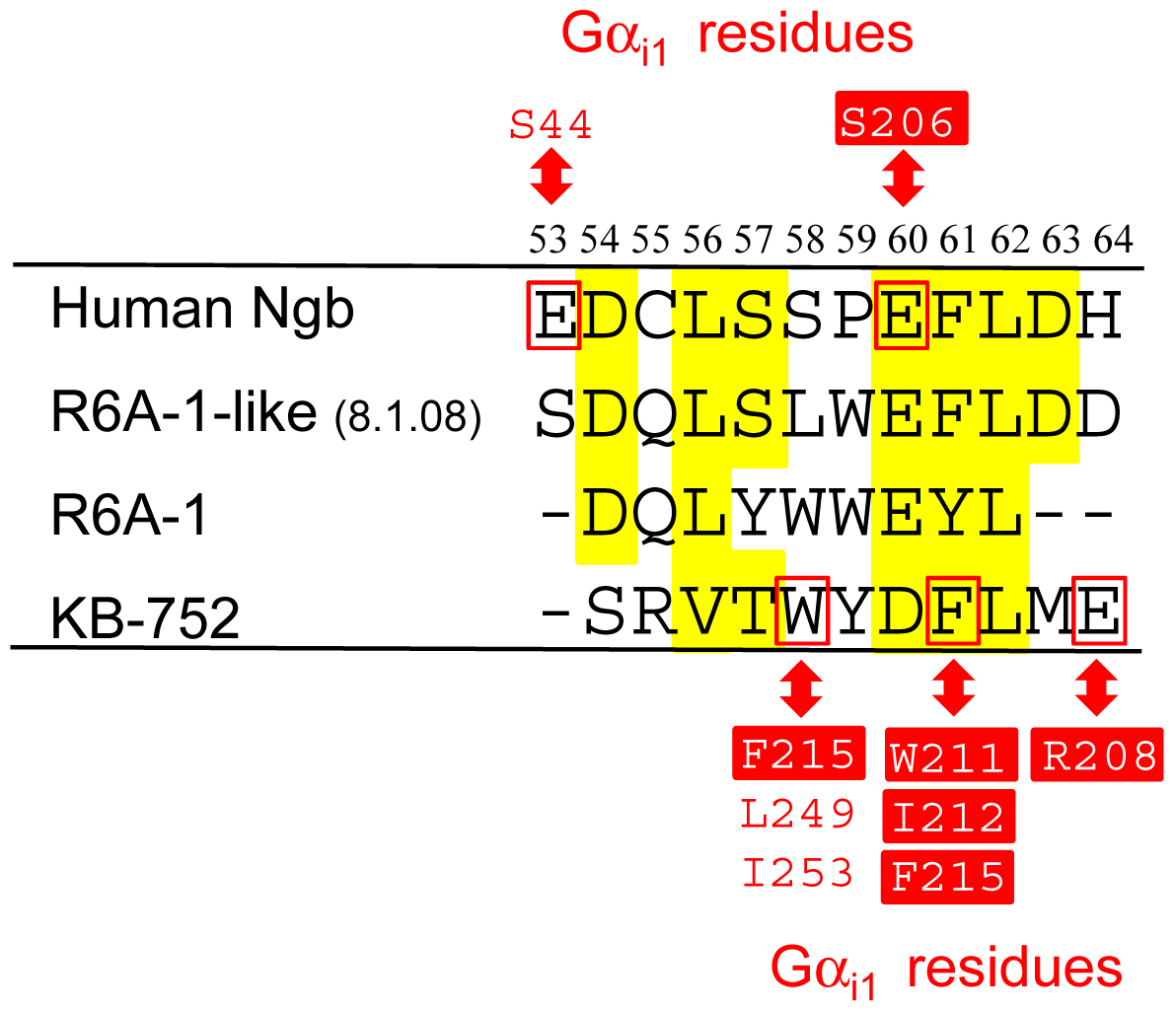
Multiple partial sequence alignment of human Ngb, R6A-1 peptide, R6A-1-like peptide (8.1.08) and KB-752 peptide. Homologous residues based on the sequence alignment among human Ngb and some peptides containing core motif for Gα binding are highlighted in yellow. Numbers above the sequences correspond to those of the residues of human Ngb. Gaps in the sequences are indicated by dashes. Binding sites in human Ngb and Gα_i1_ identified by MS analyses combined with chemical cross-linking of the proteins are listed: Glu53 and Glu60 of human Ngb are cross-linked to Ser44 and Ser206 of Gα_i1_, respectively [23]. The specific Gα_i1_ amino acid residues that interact with amino acid residues in KB-752 peptide (Trp5, Phe8, and Glu11) are listed based on the structure of the KB-752 peptide bound to Gα_i1_ [41]. Residues in the switch II (a.a. 199–219) of Gα_i1_ were highlighted in red boxes.

In the present study, we clarified the significance of Glu60 of human Ngb in terms of GDI and neuroprotective activities. It should be noted that the amino acid sequence surrounding Glu60 in human Ngb has a motif homologous to those of the R6A-1 peptide and KB-752 peptide, which interact with GDP-bound Gα_i1_ (Fig. 6). R6A peptide was isolated by *in vitro* selection using mRNA display to identify a novel peptide sequence that binds with high affinity to Gα_i1_ and was minimized to a 9-residue sequence (R6A-1) that retains high affinity and specificity for the GDP-bound state of Gα_i1_ [37]. It has previously been reported that the R6A-1 peptide interacts with Gα subunits representing all four G protein classes (Gα_i/o_, Gα_s_, Gα_q/11_, and Gα_12/13_) and binds to switch II (a.a. 199-219) of Gα_i1_ [38], [39]. R6A-1-like peptide (8.1.08; Fig. 6) was then selected from an mRNA display library of peptides based on R6A-1 [40]. On the other hand, other GDP-bound Gα_i_-binding peptides including KB-752 were identified by screening from a phage display peptide library and showed strong sequence similarity around the motif TWX(E/D)FL [41]. X-ray crystal structural determination of the Gα_i1_/KB-752 peptide complex revealed that the conserved motif in KB-752 peptide interacts with the switch II of Gα_i1_ [41]. Fig. 6 shows the partial sequence alignment among human Ngb, R6A-1 peptide, R6A-1-like peptide (8.1.08) and KB-752 peptide. As shown in Fig. 6, a hydrophobic residue (L/V) is conserved at the 4^th^ position, along with residues (E/D)(F/Y)L at the 8^th^-10^th^ positions. Because our previous MS analyses combined with chemical cross-linking of the proteins clarified that the Glu60 of the Glu-Phe-Leu motif of human Ngb is located in the switch II of Gα_i1_ like R6A-1 and KB-752 peptides [23], the corresponding region including Glu60 in human Ngb may function as the core motif for Gα binding.

In the present study, we showed that human Ngb mutants, which do not function as GDI, have almost the same hydroxyl radical scavenging activity as human WT Ngb. Moreover, we previously demonstrated that zebrafish Ngb, which does not act a GDI [27], and chimeric ZHHH Ngb, which acts as a GDI [27], have almost the same hydroxyl radical scavenging activity as human WT Ngb [32]. Because the human Ngb mutants and zebrafish Ngb cannot protect cells against oxidative stress, these results suggest that the scavenging activity of human WT Ngb against ROS is not essential for its neuroprotective activity. Moreover, although it has been reported that both human H64V Ngb and Mb generate very reactive cytotoxic ferryl (Fe^4+^) species upon treatment of the ferric form with peroxide [18], we recently showed that neither Mb nor human H64V Ngb enhanced cell death [21]. Taken together, we conclude that the neuroprotective effect of human Ngb is due to its GDI activity and not to its scavenging activity against ROS.
